# Characterization and Evaluation of CD24 and NPY as Biomarkers for Metastatic Castration-Resistant Prostate Cancer

**DOI:** 10.3390/diagnostics16050657

**Published:** 2026-02-25

**Authors:** Peter R. McHenry, Najla Fakhruddin, Kevin Homer, Rui M. Gil da Costa, Lawrence D. True, Colm Morrissey

**Affiliations:** 1 Department of Biology, Middle East University, Beirut 1202-2040, Lebanon; 2 Department of Pathology and Laboratory Medicine, American University of Beirut Medical Center, Beirut 1107-2020, Lebanon; nf62@aub.edu.lb; 3 Texas Pathology Consultants, Burleson, TX 76028, USA; 4 Division of Human Biology, Fred Hutchinson Cancer Center, Seattle, WA 98109, USA; rmcosta@fe.up.pt; 5 Department of Laboratory Medicine and Pathology, University of Washington, Seattle, WA 98195, USA; ltrue@uw.edu; 6 Department of Urology, University of Washington, Seattle, WA 98195, USA; cmorriss@uw.edu

**Keywords:** prostate cancer, CRPC, metastasis, biomarker, IHC, CD24, NPY, nuclear, AR

## Abstract

**Background/Objectives**: Prostate cancer is the most diagnosed and third most deadly cancer among men in Europe. Metastatic castration-resistant prostate cancer (mCRPC) is incurable and resistant to standard androgen ablation therapy. More biomarkers are needed to select patients for novel personalized treatments. Previous whole-genome RNA sequencing results indicated a possible role for cluster of differentiation 24 (CD24) and neuropeptide Y (NPY) as diagnostic or prognostic biomarkers in androgen receptor-positive (AR^+^) mCRPC. **Methods**: We analyzed tissue microarrays representing 127 primary prostate cancers (with matched adjacent benign prostatic glands) and 124 metastases (from 34 patients) using immunohistochemistry to detect CD24 or NPY. **Results**: CD24 was more highly expressed in primary prostate cancer than in adjacent benign tissue for nuclear (*p*: <0.001), cytoplasmic (*p*: <0.001), and membranous staining (*p*: <0.001), while NPY showed no difference. Average NPY scores were lower in prostate cancers that later recurred (geometric mean 17.6, 95% CI: 9.5–32.5) compared to those that did not (38.7, CI: 23.2–64.4; *p*: 0.044, d: 0.773). In mCRPC, CD24 was detectable in 76% of cores at the cell membrane and in 58% in the nucleus. NPY was detectable in the cytoplasm of 17%. Scores for NPY and nuclear (but not membranous) CD24 were higher in AR^+^ mCRPC. In the RNA sequencing results, *CD24* did not correlate with *AR* or *kallikrein-related peptidase 3* (*KLK3)*, while *NPY* positively correlated with *AR* (r_s_: 0.313; *p*: <0.004) and *KLK3* (r_s_: 0.400; *p*: <0.004). NPY and CD24 scores did not correlate with neuroendocrine mCRPC markers. Nuclear and membranous CD24 showed differential expression by metastatic site. **Conclusions**: We did not find strong evidence to support the use of CD24 or NPY alone as clinical biomarkers. Membranous and nuclear CD24 were expressed in the majority of mCRPC specimens, while NPY expression was more limited. NPY and nuclear CD24 were more highly expressed in AR^+^ mCRPC than AR^−^ neuroendocrine disease, and nuclear CD24 displayed site-specific expression, suggesting a potential role for nuclear CD24 in promoting AR^+^ mCRPC.

## 1. Introduction

As of 2022, prostate cancer was the most diagnosed cancer in European men and the third most deadly [1]. The current standard of care includes androgen ablation therapy, which reduces androgen receptor (AR) signaling. However, prostate cancers often develop resistance to this therapy and recur in an incurable form called metastatic castration-resistant prostate cancer (mCRPC). Second-generation antiandrogens that target the AR signaling pathways have extended survival times, but have not resulted in cures [2]. An observational study from Sweden found that median prostate cancer-specific survival from CRPC onset was 30.3 (27.5–34.1) months among patients without metastases at initial diagnosis, and 13.3 (12.1–15.8) months among patients with metastases [3].

In the hope of developing better treatment options for end-stage disease, we previously identified several novel neuroendocrine mCRPC subtypes [4] using a whole-genome RNA sequencing (RNA-seq) analysis. This RNA-seq dataset also identified two genes as highly expressed in AR^+^ mCRPC: *cluster of differentiation 24* (*CD24*) and *neuropeptide Y* (*NPY*).

CD24 is a mucin-like GPI-anchored glycoprotein. Its protein core is composed of just 31 amino acids, and its glycosylation patterns are variable. Surface-expressed CD24 is a receptor that binds to a number of different ligands and participates in many cellular activities, including protein phosphorylation, apoptosis, lipid raft formation, and transcription factor activation [5]. However, recent studies have investigated CD24 protein expression in other cellular compartments [6]. Specifically, researchers have reported the presence of CD24 in the nuclei of various types of cells, including fibroblasts and cancer cells [7,8]. CD24 has already been associated with prostate cancer. In transformed human prostate cancer cells, knock-down of CD24 reduced cell proliferation, colony formation, wound-healing, cell adhesion and invasion, and reduced tumor growth in xenografted mice. Conversely, overexpression of CD24 increased tumorigenesis and migration [9]

The second protein identified in the RNA-seq data, NPY, is a 36-amino acid secreted peptide similar to peptide YY and pancreatic polypeptide [10]. NPY is highly expressed throughout the nervous system and in the prostate gland, and mass spectrometry analyses have identified NPY as a highly overexpressed component of the prostate cancer proteome [11,12]. The precise role of NPY in prostate cancer remains to be resolved, but a study using artificial intelligence determined that NPY was one of 10 ‘hub genes’ that could serve as diagnostic or prognostic markers for prostate cancer [13].

Reliable biomarkers are important for developing new prostate cancer treatments. A recent review by Ayzman, Pachynski, and Reimers [14] discusses the promise of novel mCRPC treatments such as prostate-specific membrane antigen (PSMA)-targeted radioligand therapies and bispecific T-cell engagers but notes that tumor heterogeneity is one major challenge for effective treatment. The authors conclude that there is a need for additional biomarkers and that the future of treatment will involve a “personalized, multimodal approach,” including “strategic biomarker-driven patient selection.”

Another review by Abida, Beltran, and Raychaudhuri [15] discusses recent advances in personalized medicine to treat late-stage prostate cancer, including neuroendocrine mCRPC. The authors conclude that treatment of neuroendocrine prostate cancer “increasingly relies on genomic and phenotypic characterization of disease.” Clearly, to make progress in this area, we must discover more relevant biomarkers for use in diagnosis, prognosis, or potentially targeted therapies.

The current study had several objectives: (1) To evaluate CD24 and NPY as diagnostic biomarkers, we characterized the immunostaining of these proteins and compared their expression between primary prostate acinar adenocarcinomas and adjacent benign prostatic glands. (2) To evaluate the prognostic value of these proteins, we correlated expression levels with ISUP Grade Groups, biochemical recurrence, and other clinicopathologic parameters. (3) To explore the potential of these proteins as therapeutic targets against a subset of AR^+^ mCRPC, we compared their expression by AR status and metastatic site, and we also contrasted their expression against a profile of AR^−^ neuroendocrine mCRPC.

## 2. Materials and Methods

### 2.1. Patient Samples

Tissue microarrays (TMAs) representing 127 primary prostate cancers (and adjacent benign prostatic glands) and 124 CRPC metastases (from 34 prostate cancer patients) were obtained from the prostate cancer donor program and rapid autopsy program at the University of Washington (IRB#2341). For primary cancer specimens, patients were hormone naïve and treatment naïve at the time of radical prostatectomy. Gleason and immunostaining analyses were conducted on prostatectomy specimens. Recurrent cancer was defined as a prostate-specific antigen (PSA) level ≥ 0.2 ng/mL in two consecutive measurements. Tissues were acquired as previously described [16]. Cores from formalin-fixed paraffin-embedded tissues were used to create tissue microarray (TMA) slides for further analysis. Summaries of patient demographics can be found in Table 1 for primary cancer samples and Table 2 for mCRPC samples.

### 2.2. RNA-Seq

The RNA-Seq data that was used for this report can be accessed through Gene Expression Omnibus (GEO) at GSE126078 and GSE228283.

### 2.3. Immunohistochemistry

Immunohistochemistry (IHC) was performed as previously described [16]. Briefly, 5 µm sections of TMAs were deparaffinized and rehydrated in xylene and graded ethanol. Antigen retrieval was performed in 10 mM citrate buffer (pH 6.0) in a pressure cooker for 30 min. Endogenous peroxidase and avidin/biotin were respectively blocked (Vector Laboratories, Newark, CA, USA). TMAs were then blocked with 5% normal goat/horse/chicken serum, incubated with primary antibody in serum block overnight at 4 °C, and incubated with biotinylated secondary antibody (Vector Laboratories), followed by ABC reagent (Vector Laboratories), and stable DAB (Thermo Fisher Scientific, Waltham, MA, USA). All the sections were counterstained with hematoxylin with Scott’s bluing reagent and mounted with Cytoseal XYL (Richard Allan Scientific, Kalamazoo, MI, USA). IHC scores were calculated as the product of expression index (%) and staining intensity (0, +1, or +2), resulting in values between 0 and 200 as previously described [4]. Localization of staining was also recorded. Each stain was scored by a trained pathologist. Samples with missing or damaged cores were excluded from analysis.

SWA-11 monoclonal mouse antibody (gift from Hans-Peter Altevogt, German Cancer Research Center, Heidelberg, Germany) at a concentration of 2 µg/mL was used to stain for CD24. Secondary goat anti-mouse IgG biotinylated antibody (Vector Laboratories) was used at a 1:150 dilution. Clone 904032 monoclonal mouse antibody (R&D Systems, Minneapolis, MN, USA) at a concentration of 2 µg/mL was used to stain for NPY. Mouse IgG (Vector Laboratories) was used as an isotype control for both primary antibodies. AR, PSA, chromogranin A (CHGA) and synaptophysin (SYP) were stained as described in [17]. Staining patterns were interpreted according to expected cellular localization (AR and PSA: nuclear/cytoplasmic; CHGA and SYP: cytoplasmic/granular).

### 2.4. Statistical Analysis

Duplicate cores from prostate cancer and adjacent benign tissue were scored, and duplicates were averaged. Triplicate cores from metastatic tumors were scored and averaged.

When unstained samples were omitted from the data sets, the remaining stained sample groups were found to be log-normal. This was true for both primary tumors (Supplementary Figure S1) and metastatic tumors (Supplementary Figure S2). To avoid ignoring the unstained samples, a two-part semicontinuous approach was used. Scores of zero were assigned as ‘negative’, and scores > 0 were assigned as ‘positive’. This binary data was evaluated by a Chi-squared test for unpaired data, a two-sided binomial test for paired data, and logistic regression for associations with clinical factors.

The positive scores were analyzed as continuous data. Normality of sample data was assessed using the Shapiro–Wilk test, and equality of variance was assessed using Levene’s test. Where possible, Welch’s correction was applied to prevent Type I errors due to unequal variances. Parametric tests were used when test assumptions were satisfied. When parametric assumptions were not met, then non-parametric methods were used.

Comparisons of cancer with the adjacent benign prostate gland used a paired Student’s *t*-test. Comparisons of Gleason scores used Welch’s ANOVA with Tukey’s post-test. Comparisons of recurrent versus non-recurrent tumors were analyzed using Welch’s *t*-test.

Association of clinical factors with protein marker continuous data was assessed by multiple regression. Ten clinical variables were considered—those relating to age, time until metastasis or death, or PSA levels. Variables describing the type or duration of various treatments had few observations and were not considered. Residual analysis of continuous variables under standard linear regression showed heteroscedasticity (funnel-shaped plot), even when multicollinear predictors (condition index > 30.0) were removed. Instead, continuous variables were analyzed using a generalized linear model with a gamma distribution and log link, which are appropriate for right-skewed, non-normally distributed outcomes. When tested using our data, this model showed low deviance and Akaike information criterion (AIC) compared with other distribution models. Log transformation of concentration and time predictor variables further improved the model. Multicollinearity was detected by the variance inflation factor (VIF), and predictor variables with VIF > 5.0 were iteratively removed. Residuals were plotted versus predictors to verify that they were unbiased and homoscedastic. The fitted model was not overdispersed (residual deviance/degrees of freedom ≈ 1), and it was not statistically significantly different from the saturated model (likelihood-ratio test, *p* > 0.05). For ease in interpretation, the exponentiated beta coefficient (*e*^β^) was calculated to report the proportional change in mean score for each unit change in the independent variable. Corrected *p*-values were reported using the Bonferroni method (multiplying the uncorrected *p*-value by the number of comparisons) to control Type I errors.

Analysis of scores by metastatic site used the Kruskal–Wallis test with Dunn’s post-test. Comparisons of gene expression to protein expression and comparisons of AR^+^ to AR^−^ tumors used Mann–Whitney U test. Associations between CD24 and NPY and neuroendocrine markers were analyzed using Spearman’s rho correlation.

Means, coefficients, and effect sizes were reported with 95% confidence intervals (95% CI). Two-tailed *p*-values < 0.05 were considered statistically significant. Statistical analyses were performed using the JASP software (version 0.19.3) [18].

## 3. Results

### 3.1. Characterization of CD24 and NPY Immunostaining

To determine protein expression and localization of CD24 and NPY in prostate cancer, we immunostained sections of 127 primary prostate tumors and their adjacent benign tissue in TMA slides. Anti-CD24 and anti-NPY antibodies were used in parallel with IgG isotype control antibodies, which showed negative staining in all tumor cellular compartments and stroma.

**Figure 1:**
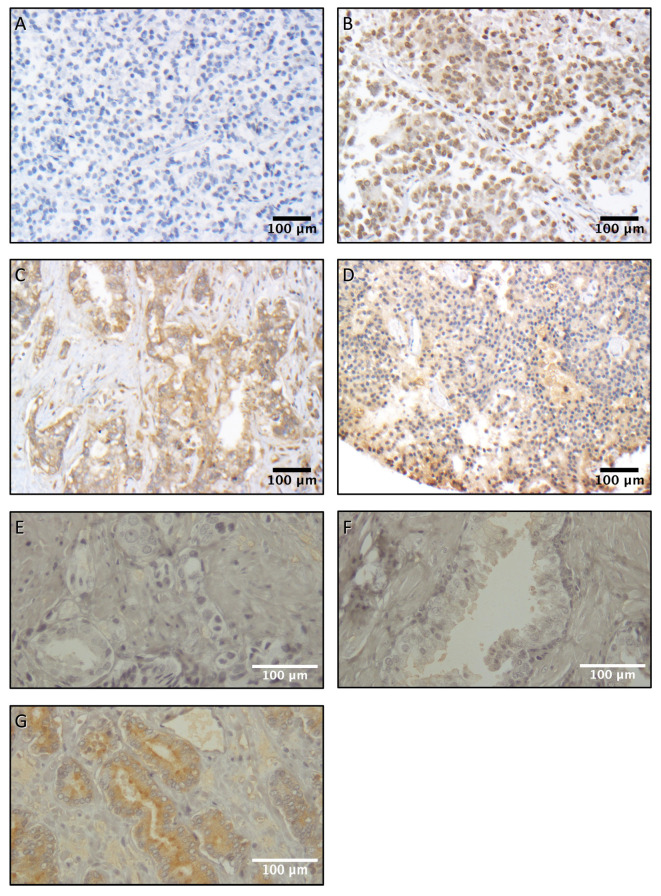
Cluster of differentiation 24 (CD24) and neuropeptide Y (NPY) staining in primary prostate acinar adenocarcinomas. Representative cores show (**A**) negative, (**B**) nuclear, (**C**) membranous, and (**D**) cytoplasmic and stromal expression. Staining Intensity was quantified as (**E**) negative/0, (**F**) low/+1, or (**G**) high/+2. (**A**–**D**) CD24, magnification 200×. (**E**–**G**) NPY, magnification 400×.

In primary prostate cancers, CD24 was detectable in the nucleus (78.3% of TMA cores), cytoplasm (77.1%), and cell membrane (35.6%) of the tumor cells and in stromal cells (34.1%). NPY was detectable in the cytoplasm (23.0% of TMA cores), cell membrane (10%), and nucleus (0.2%) of the tumor cells and in stromal cells (0.6%). Figure 1 shows representative images of CD24 and NPY staining.

In metastatic prostate cancers, CD24 was detectable only at the cell membrane (76.1% of TMA cores) or in the nucleus (58.2%). Therefore, only nuclear and membranous staining of CD24 was scored. NPY was detectable only in the cytoplasm (17.3% of the TMA cores), and only cytoplasmic staining of NPY was scored.

### 3.2. Comparison of Protein Expression Between Prostate Cancer and Normal Tissue

**Figure 2:**
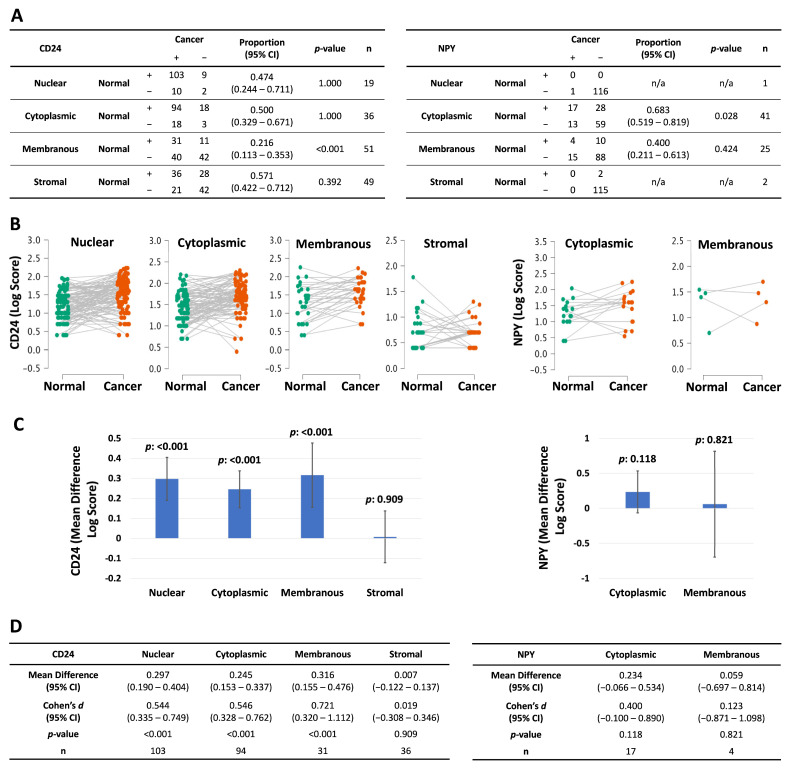
Comparison of CD24 and NPY expression in prostate cancer and normal adjacent epithelium. Protein expression was quantified (score = staining intensity (0, 1, or 2) multiplied by percent involvement): (**A**) Analysis of positive/negative staining for CD24 and NPY. The proportion of Type I discordants (Normal +/Cancer −) to total discordants (+/− and −/+) was calculated, and a 2-sided binomial test compared the proportion to the theoretical value of 0.500. (+: positive staining; −: negative staining; n/a: sample size too small to evaluate.) (**B**) Before-and-after plots comparing log-transformed scores from positive-staining cancer and normal adjacent epithelium. (**C**) Mean differences from (**B**). (**D**) The results of the paired Student’s *t*-test from (**B**). (Error bars = 95% CI.)

To determine whether CD24 protein levels were altered in prostate cancer, we compared the scores for each primary cancer to adjacent normal prostate tissue from the same patient. The proportion of samples that stained positive was lower in adjacent benign tissue than in cancer for membranous CD24 (0.216; CI: 0.113–0.353; *p*: <0.001) and cytoplasmic NPY (0.683; CI: 0.519–0.819; *p*: <0.028) (Figure 2A).

CD24 expression was approximately two times higher in prostate cancer than in the adjacent normal tissue in all the cellular compartments (nuclear, cytoplasmic, and membranous; medium effect sizes (Cohen’s d) and *p* < 0.001 for all three compartments) but not in the stroma (Figure 2B–D). This suggests that CD24 may play a role in tumor survival and growth.

To determine whether NPY expression was also altered in primary prostate cancer, we compared the NPY scores for each primary tumor to adjacent normal prostate tissue from the same patient. NPY showed no difference in expression level between cancer and normal prostate tissue in the compartments that we could assess (Figure 2C).

### 3.3. Changes in Protein Expression with Cancer Progression

To investigate whether we could observe changes in CD24 or NPY as prostate cancer progressed, we compared the nuclear, cytoplasmic, membranous, and stromal CD24 expression and cytoplasmic and membranous NPY expression to Gleason scores from the primary tumors. Nearly all of the tumors in our study were categorized in Gleason Grade Groups 1–3 (Gleason 3 + 3, 3 + 4, or 4 + 3). The groups were compared using one-way ANOVA with Welch’s correction and Tukey’s post-test. We found no statistically significant differences in either CD24 or NPY expression between each of the Grade Groups.

**Figure 3:**
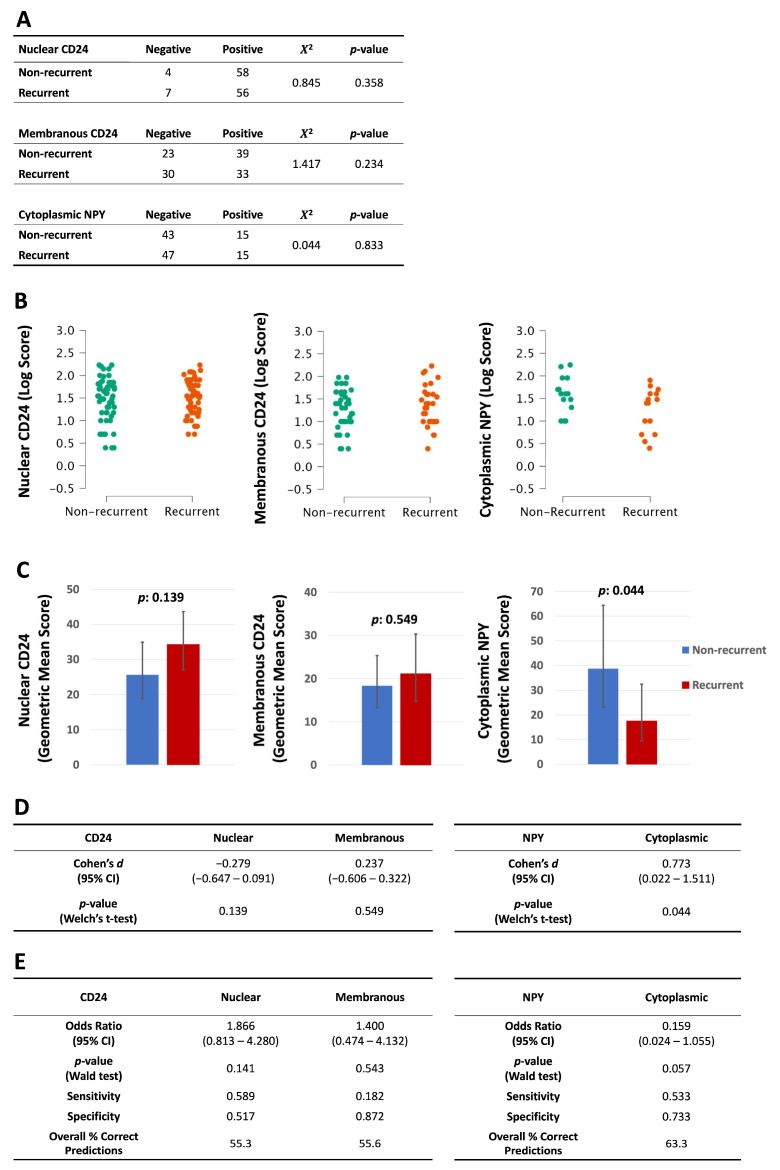
Prediction of recurrence by primary prostate cancers stained for CD24 or NPY. Prostate cancer and adjacent benign (normal) tissues were immunostained against CD24 or NPY. (Data from normal tissues was included in Supplementary Figure S3.) Binary outcome (recurrent versus non-recurrent) was determined by PSA relapse in the patient: (**A**) The Chi-squared test was used to evaluate positive versus negative staining. (**B**) Continuous data from positive-stained samples were log-transformed to approximate a Gaussian distribution. (**C**) Welch’s *t*-test was used to compare log scores on the basis of recurrence. (**D**) The results of Welch’s *t*-test for cancer samples. (**E**) Logistic regression was used to predict outcome (recurrent/non-recurrent) based on log scores from prostate cancer samples. (Error bars: 95% CI.)

To determine whether our proteins could be used to predict biochemical relapse, we compared their expression in recurrent versus non-recurrent primary prostate cancers (Figure 3). The proportion of the positive-staining cancer samples did not predict cancer recurrence (Figure 3A), although there was a statistically significant finding in adjacent normal tissue stained with CD24 (Supplementary Figure S3).

In the positively stained samples, average NPY scores were lower in the prostate cancers that later recurred (geometric mean 17.6; CI: 9.5–32.5) compared to those that did not (geometric mean 38.7; CI: 23.2–64.4; *p*: 0.044, d: 0.773) (Figure 3B–D). The CD24 scores did not change based on recurrence. To further investigate the recurrence data, we conducted logistic regression on the continuous staining data from primary tumors to measure their ability to predict PSA relapse. NPY showed a medium effect size (odds ratio (OR): 0.159, CI: 0.024–1.055) that was not statistically significant (*p*: 0.057) (Figure 3E). CD24 showed no ability to predict PSA relapse.

Others have reported differences in NPY expression ranging from a 23% increase (in expression index in prostate cancer compared with areas of perineural invasion) [19] to a 79% increase (in NPY immunofluorescence in CA1 pyramidal interneurons in constitutive androstane receptor knockout mice compared with wildtype) [20]. In comparison, the 2.2-fold difference we observed in cytoplasmic NPY expression between recurrent and non-recurrent tumors may be biologically meaningful and suggest that NPY may have a protective role against prostate cancer progression.

### 3.4. Correlation of CD24 and NPY with Clinical Outcomes in Metastatic Disease

In order to match clinical data with staining data, the scores for mCRPC samples were averaged for each patient. Essentially all the patients stained positive for nuclear and membranous CD24, but only half (17/34) of the patients stained positive for cytoplasmic NPY. Positive and negative NPY staining were approximately equal for all the clinical variables tested (Table 3A). The optimized logistic regression model fit the data well (deviance: 41.757; AIC: 45.757; dispersion: 1.35; *p*: 0.047) but had low explanatory power (McFadden R^2^: 0.087) and the tested clinical variable was not statistically significant (*p*: 0.077) (Table 3B).

Continuous data from mCRPC samples were analyzed for associations with clinical variables using a generalized linear model (GLM) with a gamma distribution and log link. A model was fitted for each stain. Certain clinical variables were log-transformed prior to analysis to improve model fit. Deviance and AIC were optimized, dispersion was monitored (<2.0), multicollinear variables were removed iteratively, and the *p*-value comparing the fitted model to a saturated model (perfect fit) showed no statistically significant difference (>0.05). For nuclear CD24 and cytoplasmic NPY, the *p*-value comparing the fitted model to an intercept-only model (baseline excluding all predictors) was statistically significant (*p*: 0.022 and 0.012, respectively) (Table 4A).

Nuclear CD24 was associated with final serum PSA (*p*: 0.048), where a 10-fold increase in PSA concentration correlated with a 51.0% increase in nuclear CD24 score. Cytoplasmic NPY was associated with survival from the first bone metastasis, where a 10-fold increase in years of survival correlated with a 10.5-fold increase in NPY score (*p*: 0.012) (Table 4B).

### 3.5. Comparison of Protein Expression at Different Metastatic Sites

**Figure 4:**
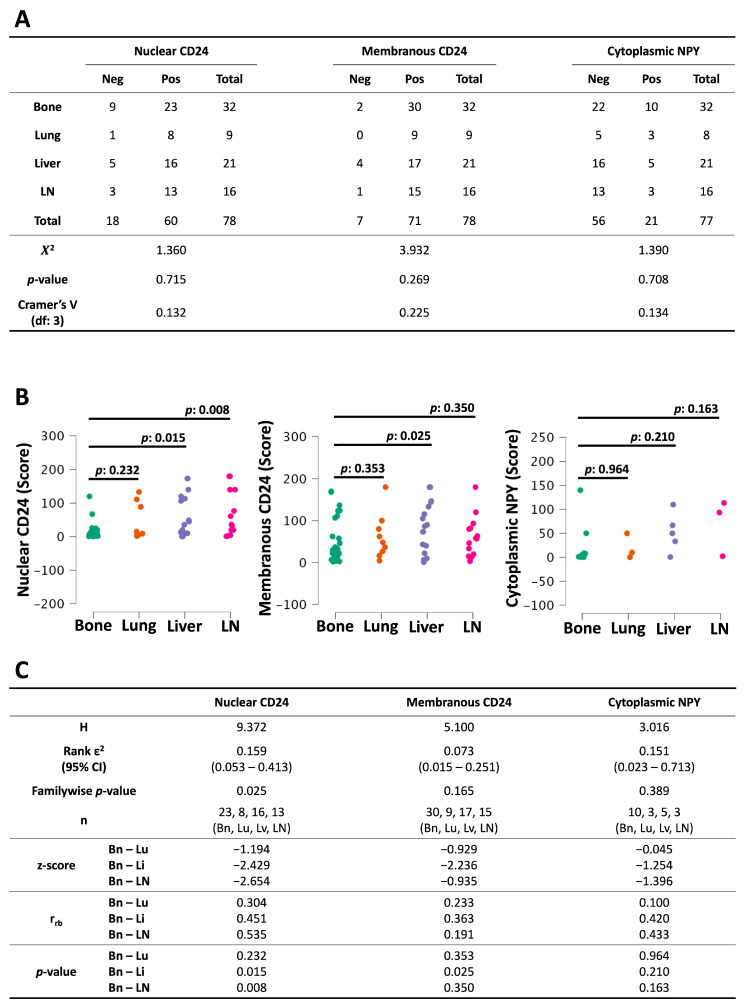
Site-specific expression of CD24 and NPY in mCRPC: (**A**) The Chi-squared test was used to evaluate positive versus negative staining for CD24 or NPY. (**B**) Continuous data from positive-staining mCRPC was compared between metastatic sites using the Kruskal–Wallis test with Dunn’s post-test to make pairwise comparisons. (**C**) The results of Kruskal–Wallis. (df: degrees of freedom; H: Kruskal–Wallis statistic; ε^2^: epsilon squared; Bn: bone (averaged per patient); Lu: lung; Lv: liver; LN: lymph node (averaged per patient); r_rb_: rank-biserial correlation.)

To investigate a potential role for CD24 or NPY in metastasis, we compared expression by metastatic site in mCRPC. We did not detect a statistically significant difference between the number of stained versus unstained samples (Figure 4A). However, median nuclear CD24 scores were lower in bone metastases compared to liver (z: −2.429; r_rb_: 0.451; *p*: 0.015) and lymph node (z: −2.654; r_rb_: 0.535; *p*: 0.008) (Figure 4B,C). Median membranous CD24 scores were also lower in bone metastases compared to liver (z: −2.236; r_rb_: 0.363; *p*: 0.025). Cytoplasmic NPY expression did not change between metastatic sites, but the comparison was limited by small sample sizes. Our data suggest there may be a role for CD24 in mCRPC that is distinct in bone compared to some visceral sites.

### 3.6. Correlation Between mRNA and Protein Expression

**Figure 5:**
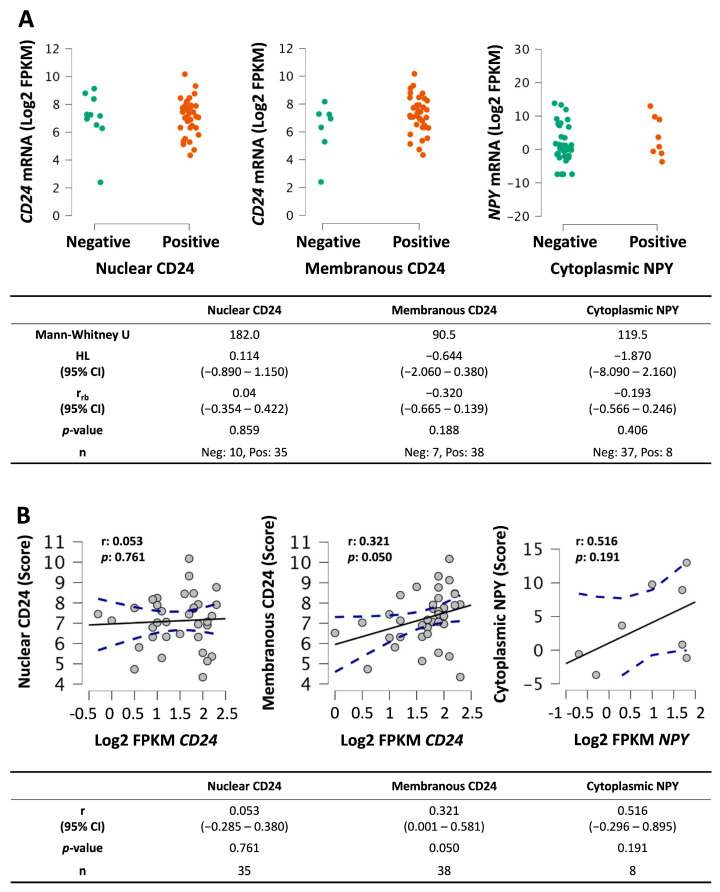
Comparison of tissue expression with gene expression of CD24 and NPY in mCRPC: (**A**) Mann–Whitney test was used to compare mRNA expression between positive and negative-staining tissues. (**B**) Continuous data from positive-staining mCRPC was tested for correlation between mRNA and tissue expression. (Log2 FPKM: fragments per kilobase of exon per million mapped fragments; HL: Hodges–Lehmann estimate; r_rb_: rank-biserial correlation; r: Pearson correlation coefficient; dotted lines in (**B**): 95% CI.)

To determine whether our previously acquired RNA-seq data correlated with the current IHC results for CD24 and NPY, we compared protein expression to RNA transcript expression from the same samples in a subset of metastases. Gene expression did not differ between negatively and positively stained mCRPC (Figure 5A). Membranous CD24 weakly correlated with transcriptional expression of the *CD24* gene (r: 0.321; *p*: 0.050), while nuclear CD24 (r: 0.053; *p*: 0.761) and NPY (r: 0.516; *p*: 0.191) did not (Figure 5B). This suggests that the usual pathway for CD24 protein expression is to traffic to the membrane, as this correlates better with gene expression values, while nuclear trafficking may represent an alternate pathway used in a subset of cells/tumors that is regulated post-transcriptionally. More experimental data are needed to clarify the mechanism of CD24 trafficking and the cellular conditions that alter localization of this protein.

### 3.7. Analysis of CD24 and NPY According to AR Status

**Figure 6:**
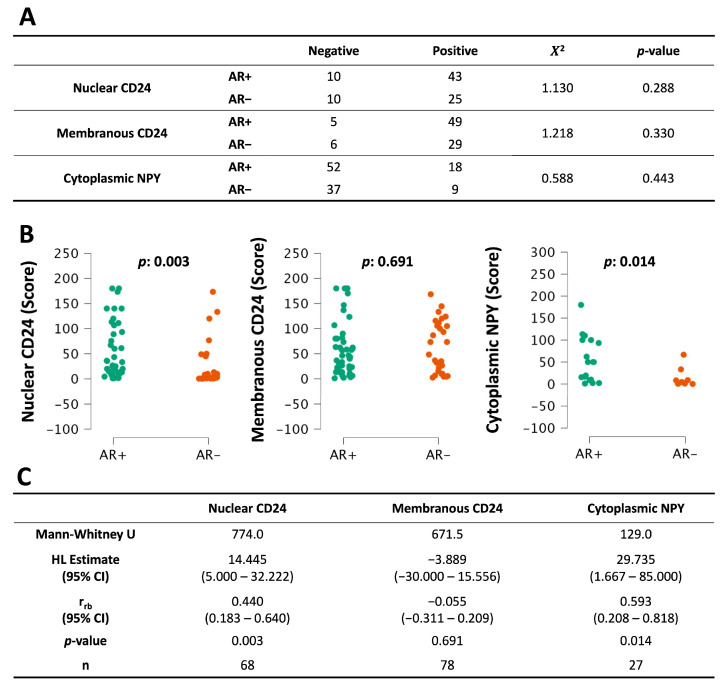
Expression of CD24 and NPY in AR-positive and AR-negative mCRPC: (**A**) The Chi-squared test was used to evaluate positive versus negative staining for CD24 or NPY. (**B**) Continuous data from positive-staining mCRPC was compared using the Mann–Whitney U test. (**C**) The results of Mann–Whitney U. AR status of these tissues was determined previously [17]. (HL: Hodges–Lehmann estimate; r_rb_: rank-biserial correlation).

AR is a primary target for prostate cancer therapy. We therefore compared CD24 and NPY expression in either AR^+^ or AR^−^ tumors. There was no statistically significant difference in the proportion of samples that stained positive for CD24 or NPY in AR^+^ mCRPC compared with AR^−^ (Figure 6A). On the other hand, AR^+^ mCRPC showed higher median scores for nuclear CD24 (HL: 14.445; r_rb_: 0.440; *p*: 0.003) and cytoplasmic NPY (HL: 29.735; r_rb_: 0.593; *p*: 0.014) (Figure 6B,C). In our RNA-seq data, however, median Log2 FPKM (fragments per kilobase of exon per million mapped fragments) values for *CD24* were similar in AR^+^ metastases and AR^−^ metastases (HL: 0.018; r_rb_: 0.008; *p*: 0.947) (Supplementary Figure S4). This suggests that, while *CD24* gene transcription does not depend on AR signaling, nuclear localization of CD24 protein may depend in part on AR. But more experimental data are needed to establish this fact.

In our RNA-seq data, median Log2 FPKM values for *NPY* gene expression were higher in AR^+^ than in AR^−^ mCRPC (HL: 7.088; r_rb_: 0.632; *p*: <0.001) (Supplementary Figure S4). Likewise, as already noted, AR^+^ mCRPC had higher median NPY scores than AR^−^, suggesting an association between these proteins. Therefore, we tested for correlations at both the transcriptional and tissue levels. We observed a weak correlation (r_s_: 0.313; *p*: <0.004) between the *NPY* and *AR* genes (Table 5), but a similar correlation was not observed between cytoplasmic NPY and AR proteins (r_s_: 0.049; *p*: 1.000) (Table 6). This suggests that there is some connection between NPY and AR, particularly at the level of transcription, but that AR is not the only driver of NPY tissue expression.

### 3.8. Correlation of CD24 and NPY with Neuroendocrine Markers

In a previous study [4], we identified several biomarkers that could be used to classify AR-positive adenocarcinoma and AR-negative neuroendocrine mCRPC. These biomarkers included AR, cellular kallikrein-related peptidase 3/prostate-specific antigen (KLK3/PSA), CHGA, and SYP. At the transcriptional level, *CD24* did not correlate with any of the phenotypic biomarkers. *NPY* was positively correlated with *AR* (r_s_: 0.313; *p*: <0.004) and *KLK3/PSA* (r_s_: 0.400; *p*: <0.004), but not correlated with *SYP* or *CHGA* (Table 5).

More importantly, we compared IHC expression of CD24 and NPY with that of the neuroendocrine markers in the same set of mCRPC. The proportion of positive-staining CD24 samples was higher in mCRPC that also stained positive for AR (OR: 1.192; *p*: <0.004) or for PSA (OR: 0.959; *p*: 0.036) (Table 6A). CD24 and NPY scores from positive-staining mCRPC showed no statistically significant correlation with any of the neuroendocrine markers (Table 6B).

## 4. Discussion

### 4.1. The Utility of CD24 and NPY as Diagnostic Biomarkers

To evaluate CD24 and NPY as diagnostic biomarkers, we first characterized the immunostaining of these proteins. The majority of patient tumors and matched normal tissues stained positive for CD24 in the various cellular compartments, while a minority of patient tissues stained positive for NPY, and expression was mainly limited to the cytoplasm.

Next, we compared their expression between primary prostate cancers and normal adjacent tissue. We found that CD24 was more highly expressed in the prostate cancers (Figure 2), suggesting a possible role in tumor survival and growth. Similarly, another study found that the percentage and intensity of CD24 IHC staining were higher in patients with prostate adenocarcinoma than in those with benign prostatic hyperplasia [21].

A liquid biopsy study showed that PSA was more useful for diagnosing prostate cancer than was secreted NPY [22]. We wondered if tissue expression of NPY would be a stronger indicator. However, we saw no difference in expression between matched normal and prostate cancer tissues. Another study quantified NPY using immunostained TMAs from prostate cancer patients. That study found that NPY and its receptors were upregulated in prostatic intraepithelial neoplasia, invasive prostate cancer, and metastases compared to benign prostate samples [19]. The expression scores in that study, however, were binned into score ranges, so comparisons with our data are challenging. Based on our results, we do not have strong evidence that NPY alone would make a useful diagnostic biomarker.

### 4.2. The Prognostic Value of CD24 and NPY

Previous studies have suggested that CD24 and NPY may serve as useful prognostic indicators. In clinical studies of prostate cancer, expression of CD24 was an early indicator of poor prognosis, PSA relapse, and decreased survival [23,24]. Nuclear-expressed CD24, specifically, was biologically active and was associated with tumorigenesis and poor patient prognosis in bladder and colorectal cancers [25]. In one clinical study, lower gene expression of *NPY* correlated with aggressive prostate tumors [26]. In another study, low *NPY* expression in a genome-wide profiling study was associated with aggressive disease and greater genomic risk, while in mCRPC in particular, low NPY was associated with neuroendocrine development [27]. In contrast, other studies suggest that NPY plays a pro-tumorigenic role in prostate cancer. For example, NPY released from murine prostate cancer cells promoted trafficking of macrophages and activation of the interleukin-6 (IL-6)/signal transducer and activator of transcription 3 (Stat3) signaling pathway in cancer cells, and this data correlated with observations from human prostate cancer patients [28].

To evaluate the prognostic value of these proteins, we compared their expression to Gleason scores in the primary prostate cancers. We found no difference in expression of CD24 or NPY when compared by Gleason score. At least one other study found that there was no correlation between NPY and tumor stage or Gleason score [29]. Another study, investigating CD24 expression, found that it increased with higher ISUP Grade Groups, especially groups 4–5 [21]. However, nearly all of our primary cancers were Grade Groups 1–3, perhaps explaining why we did not see any difference in CD24 expression.

Next, we analyzed the ability of CD24 or NPY in primary tumors to predict cancer recurrence (PSA relapse). CD24 showed no predictive ability. However, we found that median cytoplasmic NPY expression was higher in AR^+^ mCRPC (Figure 6B), suggesting an association with AR activity, although there was no direct correlation between NPY and AR in mCRPC (Table 6B). Additionally, cytoplasmic expression of NPY was lower in prostate cancers that later recurred following treatment (Figure 3C), suggesting an association with a more differentiated epithelial phenotype. Others have likewise found that NPY levels in prostate cancer cells can independently predict PSA relapse [30]. These results suggest a protective role for NPY and seem to contradict the finding that NPY and its receptors promote various aspects of prostate cancer tumorigenesis and metastasis [19]. More research is required to resolve the precise role of NPY in prostate cancer.

Finally, we analyzed CD24 and NPY expression relative to clinical factors, including serum PSA, age at diagnosis and death, survival time, and times until or following bone metastasis. Nuclear CD24 was associated only with final serum PSA, and NPY was associated only with survival time from first bone metastasis. These associations probably do not warrant the use of these proteins as standalone biomarkers for prostate cancer prognosis. In order to function as diagnostic or prognostic biomarkers, these proteins may need to be combined with other functionally correlated markers of disease, as in the case of SYP and CHGA to detect neuroendocrine tumors [31].

### 4.3. The Therapeutic Potential of Targeting CD24 and NPY

NPY is highly expressed in prostate cancer and has limited expression in other tissue types [32]. This might make it an excellent target for immune therapy in those tumors that have high expression [33,34]. Activation of NPY receptors can stimulate cell proliferation, survival, and migration [35], and thus NPY receptors have been proposed as targets for therapy against breast and prostate cancers [33]. CD24 is highly expressed in prostate cancer and has also been suggested as a target for therapy [36,37]. Similar to therapies targeting PSMA or kallikrein-related peptidase 2 (KLK2), which are in clinical use and clinical trials, secreted or membrane-associated proteins like NPY and CD24 could be targets for therapy if expressed at high levels. Researchers are already looking to CD24 as a potential therapeutic target. In urothelial and prostate cancer cells in vitro, a novel chimeric antigen receptor treatment targeting surface-expressed CD24 has already shown some promise [36].

To explore the potential of these proteins as therapeutic targets against mCRPC, we investigated their expression in metastases. Cytoplasmic NPY did not show differences, but bone metastases expressed less nuclear and membranous CD24 than some visceral sites did (Figure 4B), suggesting a role for CD24 in visceral metastasis. Although antibody performance during IHC staining can be negatively affected by bone decalcification during tissue processing, we do not believe the difference in nuclear CD24 staining is merely an artifact. We found that the proportions of stained samples were similar for all the sites, and certain visceral sites had similar scores to bone, together suggesting that the difference in nuclear CD24 reflects a real biological phenomenon.

While membrane-expressed CD24 has been extensively studied in immune signaling and cancer biology, nuclear CD24 has received less attention. We believe our data are the first to report differential expression of nuclear CD24 associated with tumorigenesis and metastasis in prostate cancer. This discovery also underscores the importance of investigating subcellular localization in histological studies. Sheng et al. demonstrated that CD24 binds to the IL-6 promoter in fibroblasts, suggesting that nuclear CD24 may function as a transcription factor [8], again highlighting the importance of studying nuclear CD24.

Next, we analyzed CD24 and NPY according to AR status in mCRPC. We found that nuclear localization of CD24 protein may be dependent on AR expression (Figure 6B), even though its transcriptional expression is not dependent on AR (Supplementary Figure S4). Membranous CD24 correlated better than the nuclear protein with *CD24* gene expression, but was not associated with AR status. Taken together, these data suggest that the typical fate for this protein may be to traffic to the membrane, correlating with transcriptional expression levels that are independent of AR, while nuclear trafficking of CD24 may represent an alternative AR-dependent pathway. Future research will investigate the cellular mechanisms of CD24 translocation into the nucleus and the role that AR may play in that process.

In contrast to CD24, NPY showed a certain degree of correlation with AR expression, especially at the mRNA level. AR-positive metastases also showed higher cytoplasmic NPY expression than AR-negative metastases, but AR does not seem to be the only driver of NPY expression. Since NPY showed variable but high expression at the protein and transcript levels in a subset of patients (AR^+^), this subset of tumors may be candidates for targeted therapy focusing on NPY, including antibody–drug conjugates or immunotherapies. The data suggest that NPY-targeted therapies would be less effective against AR^−^ (neuroendocrine) mCRPC.

Finally, we contrasted membranous and nuclear CD24 and NPY expression against a profile of neuroendocrine mCRPC. This cancer is AR^−^ but expresses CHGA and SYP. At the RNA level, *NPY* correlated with *AR* as well as *PSA* (Table 5). At the protein level, we found no statistically significant correlations with neuroendocrine markers (Table 6). However, we found that AR^+^ mCRPC expressed higher levels of both nuclear CD24 and cytoplasmic NPY (Figure 6). As such, therapies that target nuclear CD24 or NPY in mCRPC are more likely to affect tumors that are AR^+^. However, since membranous CD24 expression showed no difference based on AR status, therapy against surface-expressed CD24 could impact some patients who have AR^−^ disease for which there are no effective therapies currently.

Developing effective drugs or biologicals in this context will no doubt present a challenge to researchers. Nanomaterial strategies, exosomes, or other lipid-based drug delivery systems to deliver biologicals or miRNA may provide solutions [38,39,40]. In contrast, NPY is more easily targetable in its secreted form, and therapies that target the peptide and particularly its receptors are already under consideration [33,41].

### 4.4. Limitations of the Study

We have applied here a two-part semicontinuous model that considered negative and positive staining separately, with distinct interpretations of either binary or continuous variables. Therefore, generalizations based on the continuous data only apply to samples that stain positive for CD24 or NPY.

The majority of metastatic samples came from Caucasian donors, and no samples from African American patients were included. It is widely known that African American men are disproportionately affected by prostate cancer and underenrolled in clinical studies [42]. Additionally, risks of disease progression or death from mCRPC are different for various racial groups—even in an equal-access healthcare setting [43]. Interestingly, differential expression of CD24 in prostate tumors may partially account for racial disparities between African Americans and European Americans [44]. Unfortunately, the inclusion of mainly Caucasian men in this study limits the applicability of the findings.

## 5. Conclusions

We did not find strong evidence to support the use of CD24 or NPY alone as clinical biomarkers. Our data suggested a potential role for nuclear CD24 in tumorigenesis and progression to mCRPC. Additionally, nuclear CD24 and NPY proteins were more highly expressed in AR^+^ mCRPC than in AR^−^ neuroendocrine disease. Though protein expression was highly variable in tumors between patients, both membranous CD24 and secreted NPY may represent potential targets for therapy in patients with high tumor expression of these two proteins.

## Figures and Tables

**Table 1:** Patient demographics for primary prostate cancers.

Number of patients	127
Median age at prostatectomy (range)	59 (35–78)
Median serum PSA prior to prostatectomy in ng/mL (range)	6.3 (1.1–67.3)
Gleason score (range)	4–8
Median tumor volume in cm^3^ (range)	2.8 (0.25–9.5)
Number with positive margins	40/127 (31.5%)
Number with seminal vesicle involvement	3/123 (2.4%)
	(4 unknown)
Number of cancers that relapsed	65/127 (50.8%)
Median months to recurrence (range)	36.4 (6.0–148.3)
Median months of follow-up in non-recurrent cases (range)	81.6 (49.9–162.8)

**Table 2:** Patient demographics for metastatic castration-resistant prostate cancers (mCRPC).

**Patient Characteristics**	
Number of patients	34
Median age at diagnosis in years (range)	63.0 (43.4–77.1)
Median age at death in years (range)	67.3 (44.7–82.7)
Median survival after diagnosis in years (range)	5.4 (1.0–25.0)
Median PSA at death in ng/mL (range)	10.5 (0.7–1316.0)
Gleason score (range)	5–9
Race	31 Caucasians
	1 Pacific Islander
	1 Hispanic/Latino
	1 Unknown
**Bone Metastases/Therapies**	
Number of patients with clinically detected bone metastases	33/34 (97.1%)
Median survival after first bone metastases in years (range)	1.7 (0.2–9.2)
Number of patients receiving bisphosphonate	20/33 (60.6%)
Median treatment duration in months (range)	14.7 (1.0–108.6)
**Androgen Ablation Therapy**	
Number of patients receiving androgen ablation	34 (100%)
Median treatment duration in years (range)	4.0 (1.0–12.9)
Number of patients receiving:	
Abiraterone	8 (23.5%)
Enzalutamide	4 (11.8%)
Both	12 (35.3%)
**Other Therapies**	
Ketoconazole	7 (20.6%)
Diethylstilbestrol (DES)/estrogen	4 (11.8%)
DES and ketoconazole	2 (5.9%)
Corticosteroids	25 (73.5%)
Taxotere	26 (76.5%)
Cabazitaxel	7 (20.6%)
Taxol	5 (14.7%)
Carboplatin	12 (35.3%)
Cisplatin	2 (5.9%)
Estramustine	1 (2.9%)
Mitoxantrone	1 (2.9%)
Apalutamide (ARN-509)	1 (2.9%)
Sipuleucel-T (Provenge) vaccine	6 (17.6%)

**Table 3:** Comparison of cytoplasmic NPY staining status from mCRPC patients: (**A**) Proportion of patients with negative or positive staining, listed by clinical variable. (**B**) Logistic regression was used to predict cytoplasmic NPY staining. The optimal regression model comprised one clinical variable (Final serum PSA). No binary analysis was performed for CD24 because nearly all the patients had at least one positive-staining metastasis. (AIC: Akaike information criterion.)

(A)							
Clinical Variables			Cytoplasmic NPY				
			Negative			Positive	
Age at diagnosis			17			17	
Age at first bone metastasis			16			17	
Age at death			17			16	
Survival from diagnosis			17			17	
Bone metastasis delay from diagnosis			15			14	
Survival from first bone metastasis			16			17	
Androgen ablation—death			17			17	
Androgen independence			16			16	
Serum PSA at diagnosis			15			17	
Final serum PSA			17			16	
**(B)**							
**Cytoplasmic NPY**	**Deviance**	**AIC**	**df**	**Dispersion**	∆Χ **^2^**	***p*-Value** **(Compared to Intercept-Only Model)**	**McFadden R^2^**
Model statistics	41.757	45.757	31	1.35	3.961	0.047	0.087
**Clinical variable**	**Odds Ratio** **(95% CI)**	***p*-Value** **(Wald Test)**		**Sensitivity**		**Specificity**	**Overall % ** **Correct Predictions**
Final serum PSA (ng/mL)	0.999 (0.998–1.000)	0.077		0.750		0.588	66.7

**Table 4:** Association of clinical variables with CD24 or NPY in positive-staining samples from mCRPC patients: (**A**) Model performance for each stain. (**B**) Effects of individual clinical variables. (Generalized linear model regression with gamma distribution and log link; β: coefficient estimate; % Change: (*e*^β^ − 1) × 100%; *p*-values corrected using Bonferroni method.)

(A)							
Dependent Variable	Deviance	AIC	df	*p*-Value (Compared to Saturated Model)	Dispersion	Χ ^2^	*p*-Value (Compared to Intercept-Only Model)
Nuclear CD24	23.857	244.947	21	0.300	1.14	16.580	0.022
Membranous CD24	15.788	279.609	20	0.730	0.79	3.751	0.368
Cytoplasmic NPY	12.729	116.833	11	0.311	1.16	23.188	0.012
**(B)**							
**Nuclear CD24**				**Exponentiated** **Coefficient ** **Estimate (*e*^β^)** **(95% CI)**	**% Change**	***p*** **-Value**	**Corrected** ***p*-Value**
Androgen ablation—death (log years)				18.467 (1.446–202.958)	+1746.7	0.022	0.132
Serum PSA at diagnosis (log ng/mL)				0.785 (0.416–1.422)	−21.5	0.457	1.000
Age at diagnosis (years)				1.024 (0.966–1.082)	+2.4	0.305	1.000
Survival from first bone metastasis (log years)				1.782 (0.586–5.217)	+78.2	0.302	1.000
Final serum PSA (log ng/mL)				1.510 (1.82–2.085)	+51.0	0.008	0.048
Androgen independence (log years)				0.182 (0.058–0.592)	−81.8	0.019	0.114
**Membranous CD24**				**Exponentiated Coefficient ** **Estimate (*e*^β^)** **(95% CI)**	**% Change**	***p*** **-Value**	**Corrected** ***p*-Value**
Androgen ablation—death (log years)				1.579 (0.214–9.806)	+57.9	0.635	1.000
Serum PSA at diagnosis (log ng/mL)				0.819 (0.483–1.412)	−18.1	0.438	1.000
Age at diagnosis (years)				1.036 (0.998–1.073)	+3.6	0.070	0.420
Survival from first bone metastasis (log years)				1.179 (0.511–2.617)	+17.9	0.717	1.000
Final serum PSA (log ng/mL)				0.929 (0.728–1.174)	−7.1	0.519	1.000
Androgen independence (log years)				0.434 (0.139–1.530)	−56.6	0.132	0.792
**Cytoplasmic NPY**				**Exponentiated ** **Coefficient ** **Estimate (*e*^β^)** **(95% CI)**	**% Change**	***p*-Value**	**Corrected** ***p*-Value**
Survival from first bone metastasis (log years)				10.549 (2.452–38.823)	+954.9	0.003	0.012
Androgen independence (log years)				5.333 (0.944–38.513)	+433.3	0.080	0.32
Androgen ablation–death (log years)				1.597 (0.064–32.590)	+59.7	0.767	1.000
Age at diagnosis (years)				0.993 (0.932–1.050)	−0.7	0.836	1.000

**Table 5:** Correlation of whole-genome RNA-sequence gene expression of CD24 and NPY with biomarkers of neuroendocrine mCRPC (AR, CHGA, SYP, and KLK3/PSA). Log2 FPKM values for each gene were compared from metastatic tumor samples that corresponded to the immunostaining data set. Associations were tested using non-parametric Spearman’s rho (r_s_). (*p*-values corrected using the Bonferroni method.)

*CD24*	r_s_	*p*-Value	Corrected *p*-Value	*n*
*AR*	0.106	0.204	0.816	144
*CHGA*	0.015	0.859	1.000	144
*SYP*	0.025	0.765	1.000	144
*KLK3/PSA*	−0.018	0.832	1.000	144
***NPY***	**r_s_**	***p*** **-Value**	**Corrected** ***p*-Value**	***n***
*AR*	0.313	<0.001	<0.004	144
*CHGA*	−0.052	0.538	1.000	144
*SYP*	−0.170	0.042	0.168	144
*KLK3/PSA*	0.400	<0.001	<0.004	144

**Table 6:** Correlation of CD24 and NPY proteins with biomarkers of neuroendocrine mCRPC (AR, CHGA, SYP, and KLK3/PSA): (**A**) Binary variables were tested for associations based on negative (−) or positive (+) immunostaining using the Chi-squared test (*Χ*^2^). (**B**) Continuous variables from positive IHC staining were tested for associations using non-parametric Spearman’s rho (r_s_). (*p*-values corrected using the Bonferroni method.)

(A)									(B)			
**Nuclear ** **CD24**		**−**	**+**	Χ **^2^**	***p*-Value**	**Corrected** ***p*-Value**	**Log Odds Ratio** **(95% CI)**	**Cramer’s V** **(df: 1)**	**r_s_**	***p*-Value**	**Corrected** ***p*-Value**	***n***
AR	−	31	26	10.838	<0.001	<0.004	1.192 (0.470–1.913)	0.282	−0.074	0.580	1.000	58
	+	21	58									
CHGA	−	40	63	0.065	0.799	1.000	0.105 (−0.707–0.918)	0.022	−0.500	0.021	0.084	21
	+	12	21									
SYP	−	38	55	0.858	0.354	1.000	0.358 (−0.402–1.119)	0.079	0.032	0.870	1.000	29
	+	14	29									
KLK3/PSA	−	25	22	6.803	0.009	0.036	0.959 (0.229–1.689)	0.224	0.161	0.212	0.848	62
	+	27	62									
**Membranous CD24**		**−**	**+**	Χ **^2^**	***p*-Value**	**Corrected** ***p*-Value**	**Log Odds Ratio** **(95% CI)**	**Cramer’s V** **(df: 1)**	**r_s_**	***p*-Value**	**Corrected** ***p*-Value**	***n***
AR	−	7	50	0.233	0.629	1.000	−0.246 (−1.248–0.755)	0.041	0.060	0.630	1.000	67
	+	12	67									
CHGA	−	15	88	0.124	0.725	1.000	0.212 (−0.968–1.392)	0.030	0.387	0.038	0.152	29
	+	4	29									
SYP	−	14	79	0.287	0.592	1.000	0.298 (−0.794–1.390)	0.046	−0.037	0.829	1.000	37
	+	5	38									
KLK3/PSA	−	5	42	0.664	0.415	1.000	−0.450 (−1.539–0.639)	0.070	0.010	0.930	1.000	75
	+	14	75									
**Cytoplasmic ** **NPY**		**−**	**+**	Χ **^2^**	***p*-Value**	**Corrected** ***p*-Value**	**Log Odds Ratio** **(95% CI)**	**Cramer’s V** **(df: 1)**	**r_s_**	***p*-Value**	**Corrected** ***p*-Value**	***n***
AR	−	48	9	1.018	0.313	1.000	0.453 (−0.432–1.338)	0.087	0.049	0.848	1.000	18
	+	61	18									
CHGA	−	82	21	0.076	0.782	1.000	−0.142 (−1.148–0.864)	0.024	−0.448	0.373	1.000	6
	+	27	6									
SYP	−	71	22	2.674	0.102	0.408	−0.857 (−1.904–0.191)	0.140	−0.821	0.089	0.356	5
	+	38	5									
KLK3/PSA	−	38	9	0.022	0.881	1.000	0.068 (−0.824–0.960)	0.013	0.266	0.285	1.000	18
	+	71	18									

## Data Availability

The data presented in this study are openly available in Gene Expression Omnibus [GEO] [https://www.ncbi.nlm.nih.gov/geo/] [GSE126078 and GSE228283].

## Supplementary Materials

The following supporting information can be downloaded at: https://www.mdpi.com/article/10.3390/diagnostics16050657/s1.

## Abbreviations

The following abbreviations are used in this manuscript:

ABC	Avidin–biotin complex
AIC	Akaike information criterion
ANOVA	Analysis of variance
AR	Androgen receptor
CD24	Cluster of differentiation 24
CHGA	Chromogranin A
CI	Confidence interval
CRPC	Castration-resistant prostate cancer
DAB	3,3′-diaminodbenzidine
GPI	Glycosylphosphatidylinositol
HL	Hodges–Lehmann estimate
HRP	Horseradish peroxidase
IgG	Immunoglobulin G
IHC	Immunohistochemistry
IL-6	Interleukin 6
ISUP	International Society of Urological Pathologists
KLK2	Kallikrein-related peptidase 2
*KLK3*	*Kallikrein-related peptidase 3* (gene that encodes PSA)
Log2 KPKM	Base-2 logarithm of fragments per kilobase of exon per million mapped fragments
mCRPC	Metastatic castration-resistant prostate cancer
NPY	Neuropeptide Y
OR	Odds ratio
PSA	Prostate-specific antigen
PSMA	Prostate-specific membrane antigen
RNA-seq	RNA sequencing
r_rb_	Rank biserial correlation
Stat3	Signal transducer and activator of transcription 3
SYP	Synaptophysin
TMA	Tissue microarray
