# Supplementary figures and images for: Characterization and Evaluation of CD24 and NPY as Biomarkers for Metastatic Castration-Resistant Prostate Cancer
Source: Diagnostics (Basel). 2026 Feb 25;16(5):657. doi: 10.3390/diagnostics16050657 (PMC12984477; doi:10.3390/diagnostics16050657)

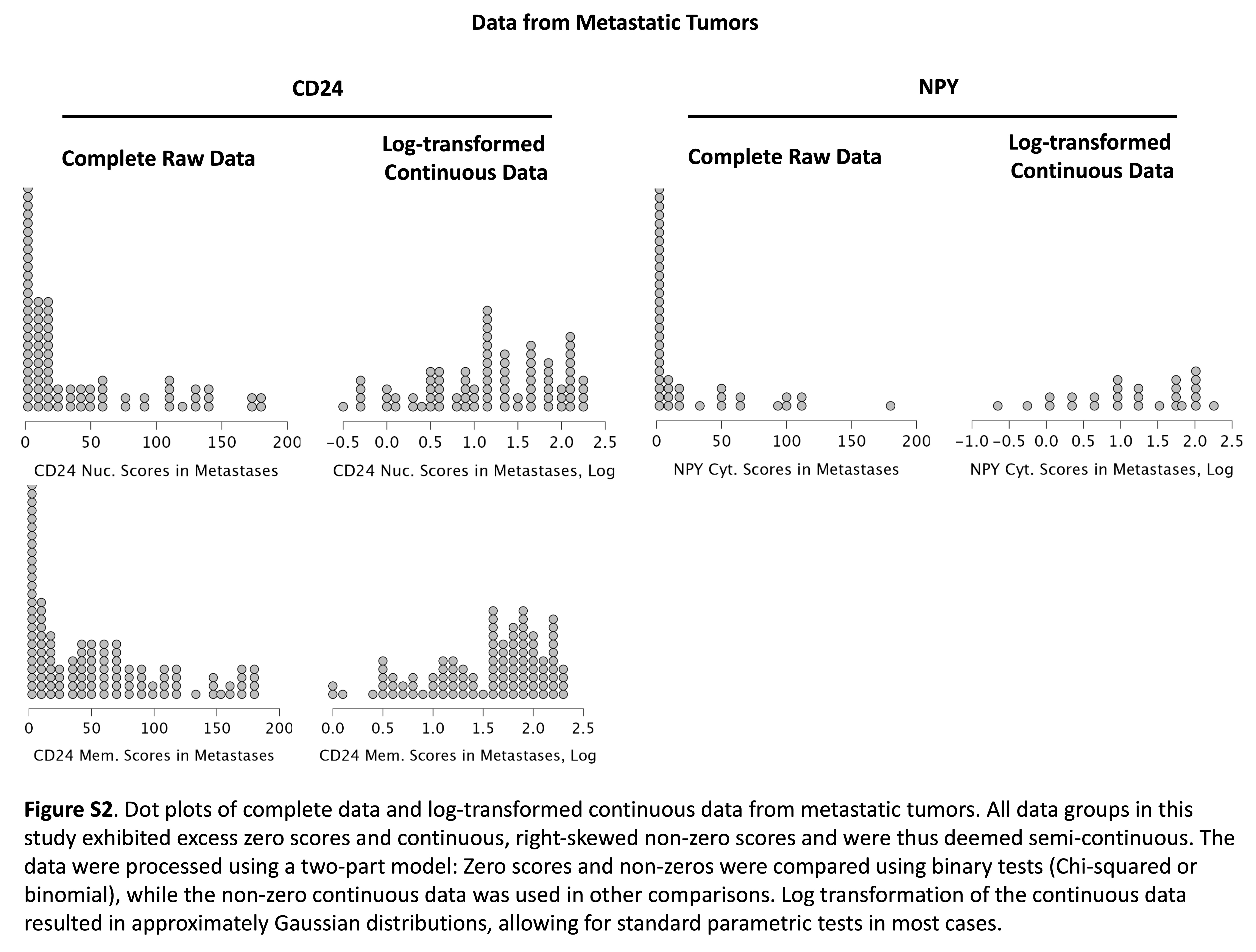

Supplement: Supplementary file 1 [file diagnostics-16-00657-s001.zip › Figure S2.jpg]

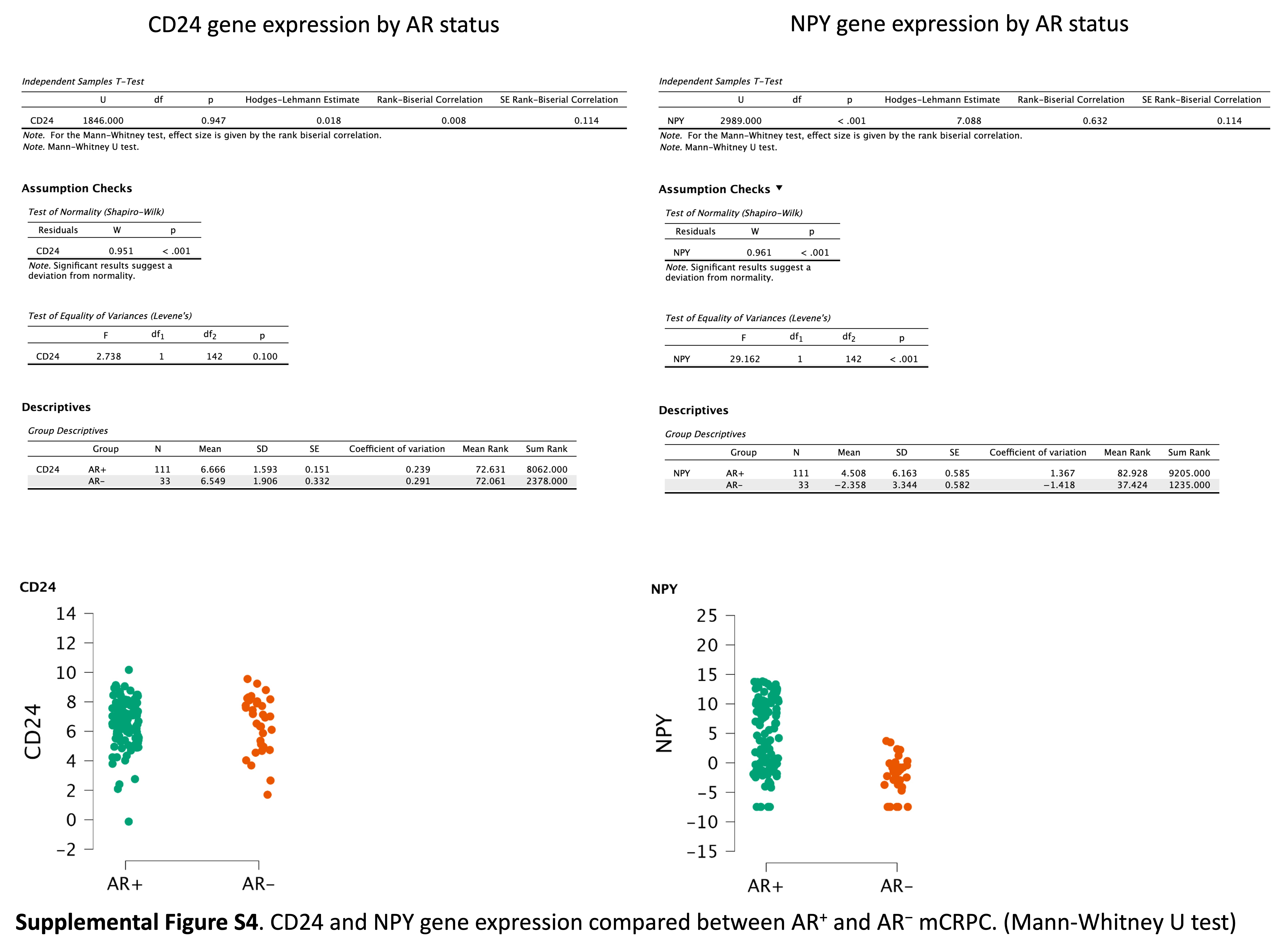

Supplement: Supplementary file 1 [file diagnostics-16-00657-s001.zip › Figure S4.jpg]
